# Type of Cooking Fuel and Mortality Among Oldest-Old in China

**DOI:** 10.1093/geroni/igaa057.363

**Published:** 2020-12-16

**Authors:** Yao Yao, Danan Gu, Huashuai Chen, Yi Zeng

**Affiliations:** 1 Peking University, Durham, North Carolina, United States; 2 United Nations, New York, New York, United States; 3 Duke University, Durham, North Carolina, United States

## Abstract

Studies have reported that elder adults are susceptible to the indoor pollution. However, evidences on the mortality risk of household air pollution (HAP) on the elderly, especially on vulnerable oldest-old population, are scarce. We aimed to estimate the association between HAP exposure from solid fuel combustion and 7-years all-cause mortality using a nationally representative dataset of oldest-old population in China. We used data from wave 2011 to wave 2018 of Chinese Longitudinal Healthy Longevity Survey. Household cooking fuel were dichotomized as solid fuel (including kerosene, charcoal, coal, wood, and biomass) and clean fuel (including electricity, solar, natural gas, and coal gas). The cohort contained 6,167 participants, totaling 21,357 person-years. There were 3,836 deaths between 2011 and 2014. About half of the participants (53.8%) used solid fuel for cooking. In the fully adjusted model, the mortality hazard ratio (HR) for solid fuel users was 1.10 (95% CI: 1.03-1.18). We observed significant interaction between HAP and urban/rural residency but not between HAP and gender. Our study showed exposure to HAP from solid fuel combustion was associated with higher risk of all-cause mortality in the male and female oldest-old population. This adverse effect was more pronounced in urban residence who use solid cooking fuels, suggesting attention should be paid on reducing HAP, particularly on susceptible population.

